# Food literacy and diet quality in young vegans, lacto-ovo vegetarians, pescatarians, flexitarians and omnivores

**DOI:** 10.1017/S1368980023002124

**Published:** 2023-12

**Authors:** Synne Groufh-Jacobsen, Christel Larsson, Wim Van Daele, Claire Margerison, Isabelle Mulkerrins, Lale Marie Aasland, Anine Christine Medin

**Affiliations:** 1 Department of Nutrition and Public Health, Faculty of Health and Sport Science, University of Agder, Universitetsveien 25, Kristiansand, Agder 4630, Norway; 2 Department of Food and Nutrition, and Sport Science, Faculty of Education, University of Gothenburg, Gothenburg, Västra Götaland, Sweden; 3 Deakin University, Institute for Physical Activity and Nutrition, Geelong, VIC, Australia

**Keywords:** Critical nutrition literacy, Diet quality, Food literacy, Flexitarian, General nutrition knowledge, Vegan, Vegetarian

## Abstract

**Objective:**

To investigate whether food literacy competencies and diet quality vary between 16-to-24-year-olds vegans, lacto-ovo vegetarians, pescatarians, flexitarians and omnivores and to assess whether food literacy is associated with diet quality.

**Design:**

Cross-sectional study. Food literacy (general nutrition knowledge, critical nutrition literacy and food skills) and diet quality were measured using an electronic questionnaire.

**Setting:**

Southern Norway, September 2021 – March 2022.

**Participants:**

Healthy 16–24-year-olds (*n* 165).

**Results:**

Overall, the mean general nutrition knowledge score was moderate (48·0 out of 67·0); the lowest mean score was found in omnivores and the highest in flexitarians (45·6 *v*. 51·5) (*P* = 0·034). The mean score of critical nutrition literacy was also moderate (3·7 out of 5·0); vegans showed higher scores compared to other dietary practices (*P* = 0·018). No difference was observed in food skills between the different dietary practices. The overall median diet quality score was 46·0 out of 80·0, lowest in omnivores and highest in vegans (42·0 *v*. 56·0) (*P* =< 0·001). In multivariate regression analyses, general nutrition knowledge, food skills and vegan dietary practice were significantly associated with higher diet quality.

**Conclusions:**

We found moderate levels of food literacy across all dietary practices. The food literacy competencies, general nutrition knowledge and food skills were associated with higher diet quality in our sample. Omnivores showed both the lowest general nutrition knowledge level and lowest diet quality scores. In contrast, both flexitarians and vegans scored highest on general nutrition knowledge and diet quality scores, despite being one of the less restrictive and one of the strictest plant-based dietary practices, respectively.

A diet consisting of ample amounts of plant-based foods is recommended for all, to avoid overuse of natural resources and to ensure a health-promoting diet^(1)^. Plant-based diets are increasingly adopted by younger people under the age of 30 years^(2,3)^. To replace animal-source foods with nutritionally equivalent food sources, one must be sufficiently food literate^(4)^.

Several definitions exist for the concept of ‘food literacy’^(5–8)^. The definition by Vidgen & Gallegos is the most cited and most widely used to date^(5,6)^, defining food literacy as: ‘…a collection of interrelated knowledge, skills, and behaviours required to plan, manage, select and prepare and eat foods to meet needs and determine food intake’^(7)^. Thus, for one to be food literate, it means being capable of navigating the current food system to make healthy food choices and to ensure food intake in alignment with the dietary guidelines^(4,7,8)^.

Youth are in a transition to adulthood and independent living; therefore, they need to be sufficiently equipped with the necessary competencies to meet their dietary requirements without the help of their parents^(9)^. For this reason, Slater *et al*.^(4)^ have proposed a broader food literacy framework for youth. Slater *et al.* suggest that ‘having more than basic nutrition knowledge’, ‘having food preparation skills’, ‘having food budgeting skills’ and ‘being able to think critically about and act on food and nutrition issues’ are especially important aspects of food literacy for youth^(4)^. In previous studies measuring food literacy in youth, none of the tools used cover the whole concept of food literacy suggested by Slater *et al.*^(10,11)^

Despite the increasing interest in plant-based diets among young people in Norway,^(2)^ studies considering food literacy in youth who follow different plant-based diets are absent^(12)^. Young people living in Norway are among those who move out from their parental housing at the youngest age in Europe,^(13,14)^ and this life phase is a critical period in which eating behaviour is being greatly influenced by peers, food marketing and media literacy^(15)^. Hence, this study was carried out to investigate if food literacy competencies and diet quality vary with vegan, lacto-ovo vegetarian, pescatarian, flexitarian and omnivore dietary practices in 16-to-24-year-olds and to assess if food literacy competencies are associated with diet quality. Thus, the hypotheses to be tested in this paper are as follows: (1) there will be no differences in food literacy competencies between those who adhere to vegan, lacto-ovo vegetarian, pescatarian flexitarian and omnivores dietary practice; (2) there are no differences in diet quality between vegan, lacto-ovo vegetarian, pescatarian, flexitarian and omnivores; and (3) there is no association between any of the food literacy competencies with diet quality.

## Methods

### Study design

This study was cross-sectional, and part of a larger mixed-methods research project called VeggiSkills Norway. The project compromises two substudies. In substudy 1, a quantitative approach was used to assess dietary intake, nutritional status, body composition, health-related behaviour and food literacy in 16-to-24-year-olds following different dietary practices. Substudy 2 used qualitative methods to explore the facilitating and inhibiting factors that shape the adoption of plant-based diets in youth. Data from parts of substudy1 are included in this study.

Data for VeggiSkills Norway were collected between September 2021 and March 2022. Healthy 16-to-24-year-olds following either vegan, lacto-ovo vegetarian, pescatarian, flexitarian or omnivores dietary practice were recruited in the Agder area in Southern Norway, through convenience sampling and the snowball sampling method. Those eligible and who consented were included in the study; thereafter, the participants completed an electronic questionnaire at the University of Agder (Kristiansand, Norway) with a researcher present. The questionnaire compromised in total 183 items assessing sociodemographic characteristics of the participants (twenty-one items), physical activity, sleeping habits and sedentary behaviour (nine items), food literacy (eighty-two items) and dietary behaviour including supplement use (seventy-one items). The questionnaire was pilot tested in two rounds before the study started.

### Study eligibility criteria

Study eligibility criteria were as follows: (1) being able to read and understand Norwegian; (2) being 16–24 years of age; (3) having no acute or chronic illness; (4) currently not pregnant/lactating; (5) not having children; (6) adherence to their respective diet for a minimum of 6 months and no current plan to alter their diet; and (7) possibility of physical attendance at the University of Agder, Kristiansand, Norway.

### Recruitment

The recruitment strategies used included physical visits to high schools, the University of Agder, private schools, folk high schools (a non-formal adult education with emphasis on experiential learning with no grades) and attendance at relevant seminars in Kristiansand. Flyers/posters were distributed in strategic places such as vegan/vegetarian restaurants/cafès. Social media recruitment was also used, including posts in closed Facebook groups and paid ads on Instagram and Facebook.

### Sociodemographic characteristics of the participants

Participant characteristics assessed included gender, age, height, weight, parental educational level, smoking status and snuff use. BMI was calculated based on objectively measured anthropometrics as weight and height (kg/m^2^).

### Classification of different dietary practices

Participants were classified into different dietary practices (vegan, lacto-ovo vegetarian, pescatarian, flexitarian, and omnivores) based on their electronic questionnaire responses. Participants were asked to report how often dairy products based on cow/goat milk, eggs and /or egg dishes, fish and/or fish dishes, poultry and meat and/or meat dishes had been consumed during the previous 6 months. If all options were reported as never and no animal-source foods were included in the diet (honey not included), participants were classified as vegans^(1)^. Participants who reported never eating fish/fish products and/or meat/processed meat but consumed milk/dairy products and/or egg/egg dishes were classified as lacto-ovo vegetarians^(1)^. Participants who additionally reported consuming fish and/or fish dishes were classified as pescatarians (regardless of reported intake of milk/dairy products/eggs or egg dishes). Participants who reported being flexitarians, defined as ‘trying to reduce their consumption of animal-source foods’ in the electronic questionnaire, and at the same time occasionally including poultry and/or meat/processed meat (<2 servings/week) in the dietary screener were classified as flexitarians^(16)^. Finally, participants who reported ‘not trying to reduce my consumption of poultry and/or meat/processed meat’ were classified as omnivores. Furthermore, participants who reported being flexitarian but reported >2 servings of poultry and meat/processed meat/week were re-classified as omnivores.

### Assessment of food literacy

Currently, no food literacy tool encompasses all the competencies proposed for young people by Slater *et al*.^(4)^. Although there are several food literacy tools available that were originally developed for adults and focus on specific food literacy competencies^(10,11,17–21)^, developing a comprehensive tool for younger age groups by adapting existing ones poses challenges. This is primarily due to the extensive range of competencies that must be incorporated and the additional requirements for suitability, accuracy and language-appropriateness needed when developing a tool tailored for younger age groups. Thus, we investigated food literacy as general nutrition knowledge, critical nutrition literacy and food skills. These three aspects were measured as these are considered important aspects of food literacy for youth in everyday practicalities for meeting dietary and nutrition recommendations^(4)^.

### Assessment of general nutrition knowledge

For the assessment of the food literacy competencies ‘having basic nutrition knowledge’^(4)^, an adapted and shortened version of the validated revised general nutrition knowledge questionnaire for adults in the UK (GNKQ-R) was used^(22)^. The adaptions made to the GNKQ-R are presented in Supplemental Table 1. Section 4 was omitted due to the discrepancy with the current Norwegian dietary guidelines for diet and prevention of disease. The adapted and shortened version of the GNKQ-R applied in this current study consists of thirty questions divided into three sections (sixty-seven items in total), with a possible maximum score of sixty-seven points, and all questions were arranged as multiple choice. For questions with multiple items, each correctly answered item gave one point.

Section 1 ‘dietary recommendations’ contained nine questions with a maximum score of eighteen points. Section 2 ‘food groups’ contained eight questions with a maximum score of thirty-six points. Section 3 ‘food choices’ contained thirteen questions with a maximum score of thirteen points. For response coding, each of the items in each question was scored as correct (1 point) or incorrect (0 points). The option ‘not sure’ was also coded as 0 points. A sum score for each section (section 1, section 2 and section 3) was calculated separately and then combined into a total sum score (total general nutrition knowledge score).

To evaluate the proportion of participants having a poor, moderate and high level of general nutrition knowledge, Bloom’s cut-off was used^(23)^. Total sum scores <60 % (<40·2 points) were considered poor, 60–79 % correct answers (40·2–52·9 points) were considered moderate, and 80–100 % correct answers (>53·6 points) were considered high.

### Assessment of critical nutrition literacy

For the assessment of the food literacy competencies ‘being able to think critically about and act on food and nutrition issues’^(4)^, a previously developed tool by Guttersrud *et al.* for measuring critical nutrition literacy was used (the CNL-C tool)^(24)^. In addition, we included a question that evaluated sources used for seeking nutrition information with response alternatives: (1) dietitian/doctor/health nurses; (2) personal trainer/dietary advisor (did not include dietitian); (3) family/friends; (4) influencers; (5) Snapchat; (6) Instagram; (7) documentaries; (8) mass media; (9) books; (10) food companies; (11) the Norwegian Health Authorities; and (12) other, if the alternative option ‘other’ was reported, an open-ended response was possible.

The critical nutrition literacy tool consists of ten items (originally eleven items in the validated tool) to measure the claims dimension of critical nutrition literacy (taking a critical stance towards nutrition claims and their sources) which have previously been validated in young Norwegian adults (see online Supplemental Table 2). Item number twenty-five from the original eleven-item tool was removed as suggested by the authors due to being a true/false statement. Each item was arranged on a five-point Likert scale with response categories (1 = disagree strongly; 2 = disagree partly; 3 = neither agree nor disagree; 4 = agree partly; 5 = agree strongly; 6 = not sure).

Four of the ten items were positive statements, meaning that agreeing to the statement equated to a higher degree of critical nutrition literacy. Response coding of positive statements was coded (1 = 1) (2 = 2) (3 = 3) (4 = 4) (5 = 5) (6 = 3). Six of the ten items were negative statements, meaning that disagreeing with the statements equated to a higher degree of critical nutrition literacy, and for these statements the scale was reversed and coded as (1 = 5) (2 = 4) (3 = 3) (4 = 2) (5 = 1) (6 = 3). For the calculation of total critical nutrition literacy score, all items were combined (positive and reversed negative statements) and subsequently divided by the number of items. The total sum score of critical nutrition literacy ranged from 1–5, of which one indicated poor critical nutrition literacy and five indicated higher critical nutrition literacy.

### Assessment of food skills

For the assessment of the food literacy competencies ‘having food preparation skills’ and ‘having food budgeting skills’^(4)^, three questions were included with predefined frequencies to assess aspects of food skills based on the domains ‘being able to develop a food budget’ and ‘being able to select healthy foods within a budget’. The questions included were as follows: (1a) ‘Do you usually do your food shopping (previous 6 months)?’; (1b) ‘If no, who usually does the food shopping for you?’ (open-ended question); (2) ‘How often do you eat pre-cooked meals outside the home or use takeaway (previous 6 months)?’; (3) ‘How often do you cook foods at home for yourself or for others (previous 6 months)?’.

To evaluate the percentage having poor, moderate, and high level of food skills, an adapted Bloom’s cut-off was used^(23)^, <60 % was considered poor, 60–79 % was considered as moderate and 80–100 % as high. For evaluation of the question: ‘how often do you eat pre-cooked meals outside the home or use takeaway?’, the cut-offs were reversed as a lower frequency of eating pre-cooked meals outside the home or using takeaway equated to higher food skills (<60 % considered high, 60–79 % was considered as moderate and 80–100 % as poor). The frequency option 2–5 times weekly was used for the food skills variable frequency of eating pre-cooked meals outside the home or using takeaway.

### Assessment of diet quality

A dietary screener (‘MinMatMåned 1·1’) was used to assess how frequently the participants consumed thirty-three selected food groups in the previous 6 months to assess alignment with the Norwegian dietary guidelines^(25)^.

The dietary screener was used to calculate a diet quality score that originally consisted of ten components (see online Supplemental Table 3) developed by Salvesen *et al.*^(26)^ The diet quality score used in this present study included eight of the ten components in the diet quality score (red and processed meat and fish components were excluded to be applicable to vegans and lacto-ovo vegetarians). To calculate the contribution of each of the components in the diet quality score, if a food was reportedly consumed 2–3 times a month, the middle value (2·5) was divided by the number of days in a month (2·5/30·5 = 0·08). The components of the diet quality score had either positive or negative scoring (based on alignment with the Norwegian dietary guidelines) ranging from 0 to 10. For a detailed scoring system of the diet quality score components, see Supplemental Table 3. Scores for each diet components were combined into a total diet quality score ranging from 0–80 points, 0 being the lowest and 80 being the highest possible score. For evaluation of diet quality, higher scores indicate a higher diet quality.

### Statistical analysis

The software used was IBM SPSS statistics version 28 (IBM Corp.). Normality was checked using a visual inspection of the histogram and Q-Q plots. One-way ANOVA with Bonferroni correction for multiple comparisons between dietary practices were used for normally distributed continuous variables and Kruskal–Wallis test for pairwise comparison for non-normally distributed variables. For categorical variables, cross-tabulation using the Fisher exact test was used. The significance level used for all tests was the *P*-value <0·05.

Multiple linear regression analysis was used to examine which of the food literacy competencies were significantly associated with diet quality score. First, univariate regression analyses were performed to examine any association between each food literacy competencies (independent variables) and the diet quality score (dependent), separately. Independent variables that were significantly associated with the diet quality score in the univariate regression analyses were retained in a preliminary model. The preliminary model was adjusted for BMI (kg/m^2^), age (years, continuous), gender (male ref.), dietary practice (omnivores ref.) and parental educational level (<16 years of education ref.). The variables adjusted for in the preliminary model were chosen based on theory. The multicollinearity of the independent variables was examined, and none were highly correlated (all *r* < 0·5).

Independent variables that were still significantly associated with the diet quality score in the preliminary model were retained in model 1. Model 1 was adjusted for BMI, age, gender and parental educational level. The final model was also adjusted for dietary practice. Interaction effects between dietary practice with the retained independent variables in the final model were examined, and no interaction effects were observed.

Multiple linear regression analysis was also used to investigate the association between participants’ dietary practice (omnivores ref.) with the diet quality score (dependent), unadjusted and adjusted for age, BMI, gender and parental educational level.

## Results

### Sociodemographic characteristics of the participants

Participant characteristics are presented in Table 1. The sample consisted of 165 participants, of which 11·5 % were vegans, 12·1 % lacto-ovo vegetarians, 18·2 % pescatarians, 15·2 % flexitarians and 43·0 % omnivores. The mean ± sd age was 21·0 ± 2·1 years, and the majority were females (75·8 %). An age difference was observed between the dietary practices (*P* = 0·010), with higher age in pescatarians compared to omnivores (*P* = 0·043), respectively. At the group level, the mean BMI was within the healthy weight range (18·5–24·9 kg/m^2^) and no differences were observed between the dietary practices. In the total sample, smoking during the previous 6 months was reported by 7·3 % and snuff use by 23·6 %; no differences were observed between the dietary practices. Half (52·3 %) of the participants reported having a parental guardian with a higher educational level (≥ 4 years of higher education); no differences were observed between the dietary practices.

**Table 1:** Sociodemographic characteristics of participants aged 16–24 years following different dietary practices (*n* 165)

Variables	All* *n* 165		Vegans* *n* 19		Lacto-ovo vegetarians* *n* 20		Pescatarian* *n* 30		Flexitarians* *n* 25		Omnivores* *n* 71		*P*-value
	*n*	%	*n*	%	*n*	%	*n*	%	*n*	%	*n*	%	
Gender†													**0·005**
Female	125	75·8	13	68·4	19	95·0	26	86·7	22	88·0	45	36·6	
Male	40	24·2	6	31·6	1	5·0	4	13·3	3	12·0	26	63·4	
Snuff use†,‡	39	23·6	7	36·8	5	25·0	7	23·3	4	16·0	16	22·5	0·621
Cigarette use†,‡	12	7·3	0	0	2	10·0	2	6·7	0	0	8	11·3	0·652
Parental educational level†													0·589
≤16 years of education	72	47·7	7	43·8	10	62·5	12	42·9	13	56·5	30	44·1	
>16 years of education	79	52·3	9	56·3	6	37·5	16	57·1	10	43·5	38	55·9	
	Mean	sd	Mean	sd	Mean	sd	Mean	sd	Mean	sd	Mean	sd	
Age, years§	21·0	2·1	21·7	2·2	20·7	1·8	21·7^ab^	1·9	21·6	1·7	20·4^cd^	2·3	**0·010**
BMI, kg/m^2^ §	23·2	3·5	22·0	2·7	23·1	4·8	23·8	3·1	22·4	3·6	23·5	3·4	0·327

* Percentage presented within each category.

† Test for difference (categorical variables) using cross-tabulation with Fisher’s exact test.

‡ Snuff use and smoking include levels rarely, occasionally and daily.

§ Test for difference (continuous variables) using one-way ANOVA with Bonferroni post hoc test correction for multiple comparisons; unlike superscript indicate differences (^ab,cd^).

Statistically significant values between the dietary practices < 0·05 are given in bold.

### General nutrition knowledge

The general nutrition knowledge scores are presented in Table 2. In the total sample, the mean total general nutrition knowledge score was 48·0 out of 67·0 points (71·6 % correct), and 30·9 % had high levels of general nutrition knowledge, 55·8 % had moderate levels, and 13·3 % had poor levels. A difference was observed between the dietary practices (*P* = 0·025), in which higher total scores were found in flexitarians compared to omnivores 51·5 (76·9 % correct) *vs*. 45·6 (68·1 % correct) points (*P* = 0·034), respectively.

**Table 2:** Food literacy competencies in youth aged 16–24 years following different dietary practices (*n* 165) in Norway

Food literacy aspects	All *n* 165		Vegans *n* 19		Lacto-ovo vegetarians *n* 20		Pescatarians *n* 30		Flexitarians *n* 25		Omnivores *n* 71		*P*-value
	Mean	sd	Mean	sd	Mean	sd	Mean	sd	Mean	sd	Mean	sd	
General nutrition knowledge score													
Section 1‡,*	13·4	2·8	13·4	4·2	13·8	2·4	13·7	2·8	14·2	2·5	13·0	2·6	0·372
Section 2‡,¶	25·1	5·2	25·8	5·1	25·8	6·1	25·9	4·6	26·9	3·6	23·7	5·5	0·061
Section 3‡,**	9·5	2·2	9·9	2·7	9·7	1·2	9·9	1·7	10·4^ab^	1·9	8·8^cd^	2·5	**0·012**
Total general nutrition knowledge score‡,§	48·0	8·8	49·1	10·9	49·2	8·8	49·5	7·7	51·5^ab^	6·2	45·6^cd^	8·9	**0·025**
Critical nutrition literacy													
Total critical nutrition literacy score‡,†	3·7	0·6	4·1^ab^	0·6	3·6	0·4	3·6^cd^	0·4	3·6^cd^	0·6	3·6^cd^	0·5	**0·011**
	*n*	%	*n*	%	*n*	%	*n*	%	*n*	%	*n*	%	
Food skills measures													
Responsible for own food shopping, always‖	109	66·1	13	68·4	13	65·0	22	73·3	21	84·0	40	56·3	0·392
Cooking of home-made food, daily‖	99	60·0	16	84·2	12	60·0	20	67·7	13	52·0	38	53·5	0·106
Eating pre-cooked meals outside the home or use of takeaway, 2–5 times weekly‖	54	32·7	3	15·8	6	30·0	8	26·7	9	36·0	28	39·4	0·371

* Section 1, dietary recommendation (maximum score = 18 points).

† Critical nutrition literacy sum score based on 10 items (claims scale) previously validated by Guttersrud *et al*.^(24)^

‡ Test for difference using one-way ANOVA with Bonferroni post hoc test, correction for multiple comparisons, significant difference indicated by unlike superscript (^ab,cd^) significance level = 0·05.

§ Total general nutrition knowledge score (maximum = 67 points).

‖ Test for difference (categorical variables) using cross-tabulation with Fisher’s exact test.

¶ Section 2, food groups (maximum score = 36 points).

** Section 3, food choices (maximum score = 13 points).

Statistically significant values < 0·05 are given in bold.

For section 1 (dietary recommendations), 68·1 % answered all correct, and for section 2 (food groups), 73·1 % answered all correct. No difference was observed between the dietary practices. For section 3 (food choices), the mean score was higher in flexitarians compared to omnivores 10·4 (80·0 % correct) *vs*. 8·8 points (67·7 %) out of thirteen points (*P* = 0·024), respectively.

### Critical nutrition literacy

The critical nutrition literacy scores are presented in Table 2. In the total sample, the mean critical nutrition literacy score was 3·7 out of 5·0, and a difference was observed between the dietary practices (*P* = 0·011). Higher levels were found in vegans compared to all other dietary practices (4·1 *vs*. 3·6). The critical nutrition literacy items were investigated separately (see online Supplemental Table 2).

Item twenty (‘I am concerned that the dietary information that I read may not be based on science’) differed between the dietary practices (*P* = 0·031). Omnivores differed from flexitarians (*P* = 0·021) and from lacto-ovo vegetarians (*P* = 0·026), with a higher percentage of omnivores reporting strongly agreeing with the statement.

Item twenty-two (‘I often refer to newspapers and magazines if I discuss diet with others’) differed between the dietary practices (*P* = 0·018). Vegans differed significantly from flexitarians (*P* = 0·044), as a higher percentage of vegans reporting strongly disagreeing with the statement.

Reported sources used for seeking nutrition information were health authorities (64·8 %), followed by documentaries/mass media and food companies (7·9 %), books (6·1 %), family/friends (5·5 %), influencers/Snapchat/Instagram (3·0 %), dietary advisor (do not include dietician) or personal trainer (2·4 %). One-tenth (10·3 %) reported using other sources when seeking nutrition information, such as Google, podcasts or YouTube, and one participant reported eating the same as people they considered as healthy individuals. No difference was observed between the dietary practices regarding which nutrition sources they used (*P* = 0·452).

### Food skills

More than half of the participants reported always being responsible for their food shopping (66·1 %) and cooking home-made foods daily (60·0 %). One-tenth reported eating pre-cooked meals outside the home or use of takeaway weekly (Table 2). No difference was observed between the dietary practices in any of the food skills items.

### Diet quality

The diet quality components and the total diet quality scores are presented in Table 3. At the group level, the median diet quality score was 46·0 out of 80·0 points. A difference was observed in the median scores between the dietary practices (*P* = <0·001). Vegans had higher score than omnivores (56·0 *vs*. 42·0, *P* = <0·001) and pescatarians (56·0 *vs*. 47·5, *P* = 0·031), respectively. Furthermore, lacto-ovo vegetarians had a higher score than omnivores (45·0 *vs*. 42·0, *P* = 0·027). Pescatarians also had a higher score than omnivores (47·5 *vs*. 42·0, *P* = 0·013). Lastly, flexitarians had higher score than omnivores (49·0 *vs*. 42·0, *P* = 0·004).

**Table 3:** Diet quality scores in youth aged 16–24 years who follow different dietary practices (*n* 165)

	All *n* 165		Vegans *n* 19		Lacto-ovo vegetarians *n* 20		Pescatarians *n* 30		Flexitarians *n* 25		Omnivores *n* 71		*P*-value
	Median	25^th^, 75^th^ §	Median	25^th^, 75^th^ §	Median	25^th^, 75^th^ §	Median	25th, 75^th^ §	Median	25th, 75^th^ §	Median	25^th^, 75^th^ §	
Vegetables*,†	8·0	6·0, 8·0	9·0^ab^	8·0, 9·0	8·0	6·0, 9·0	8·0	6·0, 9·0	8·0^ab^	6·0, 9·0	6·0^cd^	4·0, 8·0	**0·012**
Fruits and berries*,†	6·0	4·0, 8·0	8·0	4·0, 10·0	4·0	4·0, 7·5	5·0	4·0, 8·5	6·0	4·0, 8·0	4·0	2·0, 8·0	0·092
Whole grain foods*,†	8·0	6·0, 8·0	8·0	4·0, 8·0	8·0	4·5, 10·0	8·0	6·0, 8·5	8·0	8·0, 8·0	8·0	6·0, 10·0	0·754
Sugary foods*,†	6·0	4·0, 6·0	6·0	4·0, 6·0	4·0	4·0, 6·0	4·0	4·0, 6·0	4·0	4·0, 6·0	6·0	4·0, 6·0	0·758
Sugar-sweetened beverages*,†	8·0	4·0, 9·0	9·0^ab^	6·0, 10·0	8·0	6·0, 9·0	6·0	4·0, 8·3	9·0^ab^	6·0, 10·0	6·0^cd^	4·0, 9·0	**0·018**
Beans and lentils*,†	6·0	2·0, 8·0	9·0^ab,eh^	8·0, 9·0	8·0^ab^	6·0, 9·0	6·0^ab,gh^	6·0, 8·0	6·0^ab,gh^	4·0, 6·0	2·0^cd^	0·0, 6·0	**< 0·001**
Nuts and seeds*,†	2·0	1·0, 4·0	6·0^ab^	2·0, 10·0	1·0^cd^	0·0, 3·5	2·0^cd^	1·0, 4·0	4·0^cd^	0·5, 5·0	2·0^cd^	0·0, 2·0	**0·001**
Salty foods*,†	6·0	6·0, 8·0	6·0	4·0, 8·0	6·0	4·5, 8·0	6 ·0	6·0, 8·0	6·0	4·0, 8·0	8·0	6·0, 8·0	0·631
Total diet quality score*,‡	46·0	38·0, 54·0	56·0^ab,eh^	48·0, 67·0	45·0^ab^	42·0, 57·5	47·5^ab,gh^	39·8, 53·3	49·0^ab^	39·0, 56·0	42·0^cd^	34·0, 48·0	**< 0·001**

* Test for difference using Kruskal–Wallis test with pairwise comparison with Bonferroni correction for multiple tests, significant difference indicated by unlike superscript (^ab,cd,eh,gh^).

† Vegetables include salad, cabbage, carrot, green beans etc. (not potatoes or sweet potato); fruits include fruits and berries, including fresh, frozen and canned (not juice or smoothie); whole grain foods include unsweetened cereals and porridge, whole grain bread (>50 % whole grain), crisp bread, whole grain products (e.g. pasta, barley, couscous); sugary foods includes sweetened cereal and porridge, candy, including chocolate, waffles, buns, cake, biscuits, ice cream etc.; sugary beverages includes sugar-sweetened beverages and sugar-sweetened energy drinks (e.g. red bull); beans and lentils include beans, lentils, chickpeas (not green beans); nuts and seeds include unsalted nuts and seeds; salty foods include salty snacks (e.g. popcorn, chips, salty nuts).

‡ Total diet quality score includes eight components of the diet quality score combined, maximum possible points 80.

§ 25percentile, 75percentile.

Statistically significant values between the dietary practices < 0·05 are given in bold.

For the diet quality component ‘vegetables’ (*P* = 0·012), both vegans (9·0 *vs*. 6·0 omnivores, *P* = 0·002) and flexitarians had higher scores than omnivores (8·0 *vs*. 6·0 omnivores, *P* = 0·026).

For the diet quality score component ‘sugar-sweetened beverages’ (*P* = 0·018), both vegans (9·0 *vs*. 6·0 omnivores, *P* = 0·015) and flexitarians had higher diet quality scores than omnivores (9·0 *vs*. 6·0 omnivores, *P* = 0·006) with the lowest consumption among vegans and flexitarians, and highest among omnivores.

For ‘beans and lentils’, omnivores had a lower diet quality score than all other dietary practices (vegans, lacto-ovo vegetarians, and pescatarians, *P* = <0·001; flexitarians, *P* = 0·002). Vegans also had a higher diet quality score than pescatarians (9·0 *vs*. 6·0, *P* = 0·017) and higher score than the flexitarians (9·0 *vs*. 6·0, *P* = <0·001).

For ‘nuts and seeds (unsalted)’, vegans had higher diet quality score than all other dietary practices (6·0 *vs*. 1·0 lacto-ovo vegetarians, *P* = <0·001), (6·0 *vs*. 2·0 pescatarians, *P* = 0·004), (6·0 *v*. 4·0 flexitarians, *P* = 0·044) (6·0 *vs*. 2·0 omnivores, *P* = <0·001).

No differences were observed for the components ‘fruits and berries’, ‘whole grain foods’, ‘sugary foods’ and ‘salty foods’ between the dietary practices.

### Multivariate-adjusted associations

A one-unit increase in general nutrition knowledge was associated with an increase of 0·4 points in the diet quality score (*β* = 0·4, 95 % CI (0·2, 0·5)). For food skills, a 1-unit increase in the food skills variable ‘eating pre-cooked meals outside the home or using takeaway’ was associated with a decrease of 3·9 points in the diet quality score (*β* = -3·9, 95 % CI (−6·9, −0·8)) (Table 4). No other associations were observed in models examining the relationship between diet quality and independent variables.

**Table 4:** Associations between food literacy competencies and diet quality (youth aged 16–24 years, *n* 165)

	Unadjusted analysis†			Model 1*			Final model‖		
	*β* ‡	95 % CI§	*P*-value	*β* ‡	95 % CI§	*P*-value	*β* ‡	95 % CI§	*P*-value
Constant (Diet quality score)				20·81			22·59		
General nutrition knowledge	0·51	0·32, 0·69	**<0·001**	0^·^39	0·19, 0·59	**<0**^**·**^ **001**	0·35	0·15, 0·54	**<0·001**
Critical nutrition literacy	5·86	2·82, 8·90	**<0·001**						
Food skills (eating pre-cooked meals outside the home or use of takeaway)	−5·55	−8·85, −2·26	**0·001**	−4^·^71	−7·85, −1·58	**0**^**·**^ **003**	−3·87	−6·89, −0·83	**0·013**
Food skills (cooking of foods)	5·35	2·57, 8·12	**<0·001**						
Food skills (grocery shopping)	2·67	−0·09, 5·42	0·057						

* Model 1, adjusted for BMI (kg/m^2^), age (years, continuous), gender (male ref.), parental educational level (<16 years of education ref.).

† Unadjusted analysis (univariate analysis) for each of the independent variables with diet quality score (dependent variable).

‡ *β* = unstandardised *β* coefficients.

§ 95 % CI for unstandardised *β* coefficients.

‖ Final model adjusted for BMI (kg/m^2^), age (years, continuous), gender (male ref.), parental educational level (<16 years of education ref.), dietary practice (vegan, omnivorous ref), (lacto-ovo vegetarian, omnivorous ref.), (pescetarian, omnivorous ref.).

Statistically significant values < 0·05 are given in bold.

Having a vegan dietary practice was associated with an increase of 12·5 points in the diet quality score compared to having an omnivores dietary practice (reference group) (*β* = 12·5, 95 % CI (7·0, 18·0)) (Table 5).

**Table 5:** Association between dietary practice and diet quality (youth aged 16–24 years, *n* 165)

	Unadjusted analysis*			Final model‡		
	*β* †	95 % CI§	*P*-value	*β* †	95 % CI§	*P*-value
Constant (Diet quality score)				26·84		
Vegan (omnivorous ref.)	10·78	5·52, 16·04	**<0·001**	12·51	7·02, 18·01	**<0·001**
Lacto-ovo vegetarian (omnivorous ref.)	1·85	−3·54, 7·24	0·499	4·68	−0·77, 10·14	0^·^092
Pescetarian (omnivorous ref.)	1·42	−3·14, 5·98	0·540	3·47	−1·28, 8·22	0·151
Flexitarian (omnivorous ref.)	3·54	−1·34, 8·42	0·154	5·45	0·41, 10·49	0·034

* Unadjusted analysis (crude regression analysis) for each of the independent variables with the dependent variable diet quality score.

† *β* = unstandardised *β* coefficients.

‡ 95 % CI for unstandardised *β*.

§ Final model adjusted for BMI (kg/m^2^), age (years, continuous), gender (male ref) and parental educational level.

Statistically significant values < 0·05 are given in bold.

## Discussion

In this study, we investigated whether the level of food literacy and diet quality varied between 16-to-24-year-olds following vegan, lacto-ovo vegetarian, pescatarian, flexitarian and omnivores diets. We also investigated if food literacy competencies were associated with higher diet quality. An overall moderate level of food literacy across all dietary practices was found. Additionally, an overall median diet quality score of 46·0 out of 80·0 points was found. Higher level of food literacy competencies (general nutrition knowledge and food skills) was found to be associated with higher diet quality scores, along with having a vegan dietary practice. Omnivores showed the lowest general nutrition knowledge score and the lowest diet quality score. On the contrary, flexitarians and vegans had the highest general nutrition knowledge score and diet quality scores. All dietary practices showed a similar score of critical nutrition literacy, except vegans who had higher scores; however, vegans did not differ from the other dietary practices regarding which nutrition sources they used when seeking nutrition information.

### General nutrition knowledge

We found overall moderate level of general nutrition knowledge and that a higher score was associated with diet quality score. Omnivores in our study showed the lowest general nutrition knowledge score and flexitarians with the highest. Vegans, lacto-ovo vegetarians and pescatarians had similar scores. A previous study among Norwegian medical students adhering to vegetarian (*n* 95, mean age 23·5 years) and omnivore diets (*n* 299, mean age 23·6 years) reported low nutrition knowledge level^(27)^; although noteworthy, the tool used differed from the tool used in our study and was not validated to measure nutritional knowledge. Therefore, the findings of our study are not directly comparable to these results. However, a previous study measuring general nutrition knowledge based on six items adapted from the original GNKQ in vegans and vegetarians in the US (*n* 234, age 18 to 70 years and older)^(28)^ reported no difference between vegans and vegetarians, in contrast to our findings. Similarly, a study among adult respondents from Europe and Latin America, using the original GNKQ (from 1999), also did not find any difference when comparing omnivores against all dietary practices pooled in one sample (vegans / vegetarians / flexitarians)^(29)^. In contrast to these previous studies, we used a more comprehensive questionnaire for assessing general nutrition knowledge, which may partly explain the different findings. Our study sample also consisted of a younger age group.

### Critical nutrition literacy

To our knowledge, no previous studies have investigated the level of critical nutrition literacy of youth following different plant-based diets compared to a reference group of omnivores. However, a previous study reported poorer health literacy level to be associated with a pescatarian diet compared to vegan or vegetarian diets in a Canadian population (aged 16 to 30 years)^(30)^. Partly in line with our findings, the vegans in our study revealed the highest critical nutrition literacy score while lacto-ovo vegetarians, pescatarians, flexitarians and omnivores had similar scores. Another previous study among Turkish 14-to-19-year-old omnivores reported a moderate level of critical nutrition literacy^(31)^, in agreement with our findings. Another study measuring critical nutrition literacy in US students (aged 18–24 years)^(32)^ reported a mean score of 3·4, similar to our score of 3·7, using a comparable scoring system out of five (one indicated a lower level and five indicated a higher level of critical nutrition literacy). However, unlike our study, they divided the critical nutrition literacy score into three equally distributed groups to evaluate poor, moderate and high levels of critical nutrition literacy whereas we used score cut-offs to classify the groups.

### Sources used to seek nutrition information

We found discrepancies between the reported sources used to seek nutrition information and the participants own perceived abilities for evaluating nutrition information (level of critical nutrition literacy) across different dietary practices. For instance, 64·8 % of the participants reported using health authorities as a source for seeking nutrition information, while only 19·4 % strongly agreed to item 30, ‘I base my diet on information that I get from scientifically recognised literature (for instance, the journals published by the Norwegian Medical Association and the Norwegian Directorate of Health)’. Our findings might indicate that the participating youth want to be critical but lack the ability to critically evaluate the nutrition information that they are exposed to. This could potentially explain why critical nutrition literacy was not found to be associated with diet quality score, as the critical nutrition literacy items are based on their own self-perceived abilities.

### Food skills

To the best of our knowledge, studies investigating food skills and diet quality of youth following different plant-based diets compared to omnivores are lacking. We found overall moderate food skills, 60–79 % was considered as moderate. Our findings are consistent with a previous study that measured aspects of food skills in Canadian adults (mean age 22·5 years, *n* 191), in which half (47·6 %) of the participants prepared meals themselves^(33)^. Whether participants in our study were responsible for their own food shopping was included to represent the food skills competencies of food literacy ‘having food budgeting skills’ by Slater *et al.*^(4)^ Although we acknowledge that other tools may have been more suitable to measure food skills in youth, we did not include a fully validated tool to limit the participant burden.

### Diet quality

Findings in our study support previous research that youth in Norway do not consume a diet that align with the Norwegian dietary guidelines^(34,35)^. A previous systematic review found higher diet quality scores among adult vegans and vegetarians compared to omnivores when using multiple diet quality indexes^(36)^. In addition, the systematic review found a higher consumption of wholegrains and vegetables in vegans and vegetarians, and the authors suggested that animal-source foods were replaced by these food groups to some extent. Moreover, in previous research from 2002^(37)^, Swedish and Norwegian vegetarian adolescents reported a lower consumption of vegetables than expected, although vegans reported higher intakes compared to omnivores.

Interestingly, both vegans and flexitarians in our study sample were found to eat vegetables, beans and lentils more frequently and to have a lower consumption frequency of sugar-sweetened beverages compared to omnivores. While for nuts and seeds, vegans reported a more frequent consumption than all other dietary practices participating. For the diet quality score components sugary foods and salty foods, no difference was observed across the dietary practices. These findings are in line with a previous study among Norwegian adults adhering to different plant-based dietary practices^(38)^. These findings might also indicate that eating a plant-based diet does not necessarily result in a diet with lower consumption of sugary foods and salted snacks.

### Study limitations and strengths

To the best of our knowledge, this is the first study to assess food literacy in relation to a diet quality score in youth who follow different plant-based diets. Our study also had several strengths, as previously validated tools for assessing general nutrition knowledge^(22)^, critical nutrition literacy^(24)^ and diet quality (‘MinMatMåned 1·1’) were used^(26)^. The tool used for assessing general nutrition knowledge has also previously been used in young people in several countries^(39–44)^. We also adapted the validated revised GNKQ-R to the Norwegian cultural context for dietary guidelines and food choices.

However, the authors of this study acknowledge that food literacy is a complex and broad construct which can be affected by multiple factors and that it is beyond the scope of this paper to cover them all. A non-randomised design and the small sample size in each dietary practices were limitations, and the study sample consisted of predominantly females. However, previous studies investigating the dietary intake of vegans and vegetarians have also reported higher percentage females in their study population^(3,45,46)^. Regarding parental educational level, a higher percentage of the youth in our study had parents with a higher educational level compared to the general population in Norway (52·3 % *vs*. 36·0%)^(47)^, which might indicate that we have attracted a sample with higher level of socio-economic status. Having a higher socio-economic status has previously been associated with having healthier eating habits^(48)^. In our study, the diet quality score was based on a dietary screener and participants were asked to report their food consumption for the previous 6 months; thus, there is a risk of recall bias. However, dietary screener has been used in previous studies as a rapid method to assess diet quality instead of time consuming food frequency questionnaires^(49)^.

As this study was conducted during the COVID-19 pandemic, there is a risk that the participants’ reported dietary intake and food skills ‘frequency of eating pre-cooked meals outside the home or using takeaway’ may have been influenced by it. A new report by the Norwegian Institute of Public Health found that 14-year-olds who participated in the Norwegian mother and Child Cohort did not change their diet prior to, during or after the COVID-19 pandemic, except adolescents who were already consuming a higher sugar intake than recommended, they reported an additional increase in sugary foods and sugary beverages^(50)^. This present study was conducted near the end of the pandemic, there are uncertainties regarding the extent to which the pandemic has affected the participants dietary intake and reported food skills.

## Conclusions

In this study of food literacy competencies and diet quality in 16-to-24-year-old vegans, lacto-ovo vegetarians, pescatarians, flexitarians and omnivores, we observed moderate food literacy levels and suboptimal diet quality across various dietary practices. Still, there were differences across different food literacy competencies and diet quality between the dietary practices. Omnivores showed the lowest level of general nutrition knowledge and flexitarians the highest, whereas vegans demonstrated the highest level of critical nutrition literacy compared to the other dietary practices. Our findings also showed that a vegan dietary practice was associated with highest diet quality score, while omnivores had the lowest diet quality score. Furthermore, we noted a correlation between food literacy competencies (general nutrition knowledge and food skills) and diet quality in this study.

Based on these findings, we propose that further research should trial interventions aimed at specifically enhancing general nutrition knowledge and food skills competencies in youth, regardless of dietary practice. By doing so, we may improve the diet quality of young individuals, by enabling them to navigate today’s complex food landscape more effectively.

## Supporting information

Groufh-Jacobsen et al. supplementary material Groufh-Jacobsen et al. supplementary material

## Supplementary material

For supplementary material accompanying this paper visit https://doi.org/10.1017/S1368980023002124
