# Supplementary material for: Food literacy and diet quality in young vegans, lacto-ovo vegetarians, pescatarians, flexitarians and omnivores
Source: Public Health Nutr. 2023 Oct 2;26(12):3051–61. doi: 10.1017/S1368980023002124 (PMC10755439; doi:10.1017/S1368980023002124)
Supplement: Groufh-Jacobsen et al. supplementary material [file S1368980023002124sup001.docx]

**Supplementary table 1** Changes made to the revised general nutrition knowledge questionnaire for UK adults by Kliemann, N., et al. 2016.

| Changes to the revised general nutrition knowledge questionnaire (GNKQ-R) |
| --- |
| **Translation:**  For translation, the following steps were employed; 1) Adaptation of the original GNKQ-R for the target country and target population; 2) Forward translation into Norwegian; 3) Expert panel; 4) Backtranslation into English; 5) Pilot test (n = 15, includes pilot 1 and 2).  The translation of the questionnaire into Norwegian was performed by a translator familiar with the terminology of the area covered by the GNKQ-R. Subsequently, an expert panel consisting of three contributors, including the original translator, identified, and resolved inadequate expressions of the translation, as well as any discrepancies between the forward translation and the previous versions of the questionnaire. The translation of the questionnaire from Norwegian to English was carried out by an independent translator with no previous knowledge of the GNKQ-R questionnaire. The independent translator gave feedback on the conceptual equivalence and if there were any discrepancies with the changed version of the questionnaire before forward translation. Finally, the original translator changed the forward-translated version until an agreement was reached. |
| **Introduction:**   - In the VeggiSkills-Norway project, the electronic questionnaire was completed with supervision, and therefore the following parts of the description were removed: “It is important to complete it yourself. Your responses will remain anonymous.” |
| **General amendments:**   - To reduce the length, all the headings of the following sections were removed: “Section 1. Dietary recommendations”; “Section 2. Food groups”; “Section 3: Food choices”. - 'Experts' was changed to 'The Norwegian Dietary Guidelines/ Norwegian Health Authorities' throughout for the use of the more familiar terms in a Norwegian context. - 'Tick one box per food' was changed into 'tick one row' and changed throughout for consistency. - All question numbers were omitted, as the section headings were removed. - The questionnaire was completed electronically with mandatory answering, so only one alternative answer was possible. Thus, 'tick one' was omitted throughout. |
| **Section 1. Dietary recommendations** |

| - Question 1: 'Do Norwegian health authorities recommend that people eat more, the same amount, or less of the following foods? The word “drinks” was added to the question. - Question 1: All answer alternatives were altered according to the Norwegian dietary guidelines, for example: “water” changed to “water as a beverage”; “processed red meat” changed to “red meat and processed meat”, the answer alternative “salty foods” was changed into “salt and foods containing high amounts of salt”. The answer alternative “fatty foods” was removed and replaced, as currently, no Norwegian recommendation exists to reduce intake of “fatty foods”. Instead, an option was included for “Fish and fish products”. - Question 1: The frequency alternatives “more”, “less”, and “not sure” was altered into “more of”, “same amount”, “less off”, and “not sure”, for consistency with the wordings in the Norwegian dietary guidelines. - Question 2: “How many servings of fruit and vegetables per day do experts advise people to eat as a minimum?” was slightly reworded into “How many servings of fruit and vegetable are recommended daily?”, a country-specific alteration. - Question 3: “Which of these types of fats do experts recommend that people should eat less of”, was slightly reworded into “What do the Norwegian health authorities recommend for the following types of fat”. Additionally, examples were added for all types of fats. The item “trans-fat” was replaced with “saturated fats”, a country-specific alteration. The alternative saturated fat was divided into two alternatives “saturated fats from plants” and “saturated fats from animal source foods”. - Question 4: “Dairy foods” was reworded into “Milk and dairy products”, for more consistent language with the Norwegian dietary guidelines. - Question 5: “Oily fish” was slightly altered into “Fish”, as the Norwegian dietary guideline for fish includes both fatty and lean fish. The frequency alternatives were also changed accordingly, as recommended fish intake in Norway is 2-3 times a week. - Question 6: Currently, no Norwegian dietary guidelines exist for several alcoholic beverages, thus this question was removed and replaced with a question concerning wholegrain foods, a country-specific alterations. The new replacement question was as follows: “Which of the following alternatives will cover daily intake of wholegrain?” - Question 7: Currently, no Norwegian dietary guideline for eating breakfast exists. Thus, the question was removed and replaced with a question specifically for the Norwegian dietary guidelines for having a varied diet: “What does the Norwegian health authorities recommend”, with the answer alternatives 1) consume less energy than used 2) consume more energy than used 3) consume a varied diet 4) unsure. - Question 8: To reduce the length, “If a person has two glasses of fruit juice in a day” was omitted from the question, the following was used: “How many portions of fruit and vegetables does two glasses of juice count as?” - Question 9: Currently, a “eat well guide” does not exist in Norway, thus this question was removed and replaced with a country-specific question: “If foods are labeled with ‘Nøkkelhullsmerket`, these foods are compared to similar foods in the same food category without this labeling, with the alternatives, 1) more ecological 2) more sustainable 3) fair trade products 4) healthier 5) not sure. |
| --- |
| **Section 2. Food groups** |
| - Question 1: “Do you think these foods are….”, was slightly altered into “Which of the following foods are”. - Question 2: Certain food items were reworded into country-specific foods, e.g. “Canned soup” into “tomato soup (toro)” and “Baked beans” into “sausages”. The option “bread” was also altered into “industrially produced bread”, to avoid misunderstanding and reporting of homemade bread. - Question 3: “Do you think these foods are….” was slightly reworded Into, “Which of the following foods are”. The option “pasta” was slightly altered into “white pasta”, to avoid misunderstanding. - Question 4: “Do you think these foods are….” was slightly reworded into, “Which of the following foods are”. Additionally, the alternative ‘baked beans’ was altered into ‘beans and lentils, country-specific alteration. - Question 5: The option “plantains” was changed to ‘banana’. - Question 6: The option “cholesterol” was omitted, consequently “eggs” as the answer alternative was removed and replaced with answer alternatives “salmon”, “coconut oil”, and “processed and red meat” for more similarity with the Norwegian dietary guidelines. - Question 7: “Trans-fat” was altered into “saturated fat”, as trans-fat content in Norwegian products has been reduced. The answer alternatives were changed accordingly, “Biscuits, cakes and pastries” into “red meat and processed meat products”, one of the major sources of saturated fat in the Norwegian diet. - Question 9: “Which one of the following nutrients has the most calories for the same weight of food?” was slightly reworded into, “Which of the following has the most energy per 100 grams”. The word “roughage” was removed and replaced with a description of fiber, as fiber is a more familiar term for the target population group, adolescents. - Question 10: To avoid misunderstanding standing, “Higher in calories” was reworded into “ultra-processes food typically contains additives”, an alteration specific to the target population. |
| **Section 3: Food choices** |
| - Question 1: “If a person wanted to buy a yogurt…..”, the beginning of the question was omitted, to obtain simple and short questions. Additionally, the answer alternative “creamy fruit yogurt” was altered to “yogurt with prunes”. - Questions 2, 3, 4, and 5: The answer alternatives were altered into foods more commonly consumed in Norway. - Question 6: The options “red pepper, tomatoes, lettuce” was altered into “cucumber, corn, and lettuce”, to avoid misunderstanding, and for use of vegetables more commonly consumed by Norwegian adolescents. - Question 7: “If a person wanted to reduce the amount of fat in their diet, but didn`t want to give up chips, which of the following foods would be the best choice”, the following question was removed and replaced with the question, “Which of the following alternatives will be the best choice for a person who wishes to reduce their sugar intake?”, country-specific alteration. - Question 9: The option alternative “sautéing” was altered into “Wokking”, a more familiar term by adolescents in Norway (changed after the pilot test). - Question 10: Currently, traffic lights are not used on food labeling in Norway, thus this question was altered into using a country-specific food labeling “the bread scale” (“brødskalaen”). The bread scale is labeling applied in Norway to help consumers choose bread containing higher amounts of fiber. - Questions 11, 12, and 13: The product labels were replaced with two Norwegian food products, and the questions were altered according to the new food labels. |
| **Section 4: Diet and disease** |
| - Section 4 “Diet and disease” was omitted, due to a mismatch with the current Norwegian dietary guidelines for diet and prevention of disease. |
| **Section 5: Background** |
| - Section 5 “background” was replaced with a country-specific background question relevant to our project aim and target population group. |

**Supplemental table 2** Critical nutrition literacy items in 16-to 24-year old’s who follow different dietary practices (n = 165).

| Items ^¶^ | Disagree strongly | | Disagree partly | | Neither agree nor disagree | | Agree partly | | Agree strongly | | Not sure | | p-value ^‡^ |
| --- | --- | --- | --- | --- | --- | --- | --- | --- | --- | --- | --- | --- | --- |
|  | n | % | n | % | n | % | n | % | n | % | n | % |  |
| I have confidence in the various diets that I read about in newspapers, magazines, etc. (original item 24) | 70 | 42·4 | 62 | 37·6 | 23 | 13·9 | 7 | 4·2 | 0 | | 3 | 1·8 | 0·450 |
| I am critical of the dietary information that I receive from various sources in society (original item 21) | 8 | 4·8 | 10 | 6·1 | 16 | 9·7 | 71 | 43·0 | 58 | 35·2 | 2 | 1·2 | 0·164 |
| I am concerned that the dietary information that I read may not be based on science (original item 20) | 2 | 1·2 | 2 | 1·2 | 14 | 8·5 | 39 | 23·6 | 105 | 63·6 | 3 | 1·8 | **0·031** |
| I am confident that the media’s presentation of new scientific findings concerning a healthy diet is correct (original item 29) | 16 | 9·7 | 60 | 36·4 | 37 | 22·4 | 36 | 21·8 | 11 | 6·7 | 5 | 3·0 | 0·896 |
| I am familiar with the criteria for scientifically based content in health claims (original item 23) | 11 | 6·7 | 25 | 15·2 | 44 | 26·7 | 49 | 29·7 | 22 | 13·3 | 14 | 8·5 | 0·395 |
| I often refer to newspapers and magazines if I discuss diet with others (original item 22) | 87 | 52·7 | 45 | 27·3 | 14 | 8·5 | 9 | 5·5 | 5 | 3·0 | 5 | 3·0 | **0·018** |
| I am influenced by the dietary advice that I read about in newspapers, magazines, etc. (original item 26) | 58 | 35·2 | 48 | 29·1 | 18 | 10·9 | 34 | 20·6 | 3 | 1·8 | 4 | 2·4 | 0·064 |
| I am confident that some of the methods within alternative medicine (such as health foods) provide me with credible dietary advice (original item 27) | 34 | 20·6 | 32 | 19·4 | 47 | 28·5 | 31 | 18·8 | 11 | 6·7 | 10 | 6·1 | 0·212 |
| I find it hard to distinguish scientific nutritional information from non-scientific nutritional information (original item 28) | 19 | 11·5 | 41 | 24·8 | 29 | 17·6 | 50 | 30·3 | 18 | 10·9 | 8 | 4·8 | 0·285 |
| I base my diet on information that I get from scientifically recognized literature (for instance, the journals published by the Norwegian Medical Association and the Norwegian Directorate of Health) (original item 30) | 12 | 7·3 | 26 | 15·8 | 30 | 18·2 | 58 | 35·2 | 32 | 19·4 | 7 | 4·2 | 0·861 |

^¶^ n (%) presented for all dietary groups pooled in one sample (vegans, lacto-ovo vegetarians, pescatarians, flexitarians, omnivorous), the critical literacy items were developed by Guttersrud et al., 2014; ^‡^ Test for the difference between the dietary groups, the continuous score variables was used, using one-way ANOVA for items 21-24, 26-30, for item 20 (non-normally distributed) Kruskal Wallis test was used with Bonferroni correction for pairwise comparison (item 20: omnivorous differed from flexitarians (*p* = 0·021), and lacto-ovo vegetarians (*p* = 0·026); item 22: vegans differed from flexitarians (*p* = 0·044); item 26: vegans differed form flexitarians (*p* = 0·048)); Statistically significant values < 0·05 are given in bold.

| Diet score component  Screener variable(s)^¶^ | Scoring | Criteria for min score (0) | Criteria for max score (10) |  | | | | | | | | | |
| --- | --- | --- | --- | --- | --- | --- | --- | --- | --- | --- | --- | --- | --- |
|  |  |  |  | Never | once a month | 2 - 3 times a month | once a week | 2 - 4 times a week | 5 - 6 times a week | once day | 2 - 3 times a day | 4 - 5 times a day | ≥6 times a day |
| Vegetables^‡^ | positive | ≤1 x month | ≥4 x day | 0 | 0 | 1 | 2 | 4 | 6 | 8 | 9 | 10 | 10 |
| Fruits and berries^‡^ | positive | ≤1 x month | ≥2 x day | 0 | 0 | 1 | 2 | 4 | 6 | 8 | 10 | 10 | 10 |
| Whole grain (products)^‡^ | positive | ≤1 x month | 2 - 5 x day | 0 | 0 | 1 | 2 | 4 | 6 | 8 | 10 | 10 | 8 |
| Sugar-sweetened beverages^‡^ | negative | ≥1 x day | 0 | 10 | 9 | 8 | 6 | 4 | 1 | 0 | 0 | 0 | 0 |
| Sugary foods^‡^ | negative | ≥1 x day | 0 | 10 | 9 | 8 | 6 | 4 | 1 | 0 | 0 | 0 | 0 |
| Beans and lentils^‡^ | positive | 0 | ≥2 x day | 0 | 1 | 2 | 4 | 6 | 8 | 9 | 10 | 10 | 10 |
| Nuts and seeds (unsalted)^‡^ | positive | 0 | 1 - 3 x day | 0 | 1 | 2 | 4 | 6 | 8 | 10 | 10 | 8 | 6 |
| Salty foods^‡^ | negative | ≥2 x day | 0 | 10 | 9 | 8 | 6 | 4 | 2 | 1 | 0 | 0 | 0 |

**Supplemental Table 3** Diet quality score scoring system based on MinMatMåned food screener retrieved developed by Salvesen, L and coworkers, 2023.

^¶^The original diet quality score by Salvesen, L., and coworkers 20203, consisted of 10 components with 100 possible total points. In the applied modified version, animal-source food components were removed (meat and fish), thus total possible maximum points were 80; ^‡^Vegetables includes salad, cabbage, carrot, green beans etc. (not potatoes or sweet potato); ^‡^Fruits includes fruits and berries, including fresh, frozen, and canned (not juice or smoothie), Whole grain foods includes cereal and porridge, unsweetened, whole grain bread (>50% whole grain), crisp bread, whole grain products (pasta, barley, couscous, e.g.), Sugary foods includes sweetened cereal and porridge, candy, including chocolate, waffles, buns, cake, biscuits, ice cream etc.; ^‡^Sugary beverages includes sugar-sweetened beverages and sugar-sweetened energy drinks (e.g., red bull), Beans and lentils includes beans, lentils, chickpeas (not green beans), Nuts and seeds includes unsalted nuts and seeds, Salty foods includes salty snacks (e.g., popcorn, chips, salty nuts).
